# Therapy

**Published:** 1881-07

**Authors:** 


*Therapy.*—Some one has suggested the use of Eucalyptus oil and Iodoform in dressing root canals, and the writer is pleased to say that it seems a good method. Wet the cotton on the broach with the oil, and dip in the pulverized Iodoform, using as any other dressing. The oil is fragrant in smell, not unpleasant, and non-irritating. So far we have had no sore teeth that could fairly be attributed to either of these drugs. The Iodoform is alterative and the oil deodorizing and perhaps directly medicinal.

